# Supplementary material for: Trypanosoma cruzi DNA Polymerase β Is Phosphorylated In Vivo and In Vitro by Protein Kinase C (PKC) and Casein Kinase 2 (CK2)
Source: Cells. 2022 Nov 21;11(22):3693. doi: 10.3390/cells11223693 (PMC9688435; doi:10.3390/cells11223693)
Supplement: Supplementary file 1 [file cells-11-03693-s001.zip › PKC.FigureS1.pdf]

clustalw.aln

```
CLUSTAL 2.1 multiple sequence alignment


BAU98542.1      -----MADVSPANDSTASQDVANRFARKGALRQKNVHEVKDHKFIARFFKQPTFCSHCTD
KAI4054112.1    -----MADPAAGPPPSEGEESTVRFARKGALRQKNVHEVKNHKFTARFFKQPTFCSHCTD
XP_809943.1     ------------------------------------------------------------
XP_845346.1     ------------------------------------------------------------
PWV04907.1      ----------MDTVDPRGTQGESENNGNNNNNGNGNGKEGGGGSQAKATSEKNVETVRRV
ESS64742.1      MISKLFRLDPQRREKHEINPHDIKALRGMAATHNITSVAVPQYRVRENYVEYVIECSKRS
AAK64304.1      MLSKLFRYDTKLQGKQELSTADHRSLLELSMTDNITSVEIPQYRVRGTFVENVIECTKRN

BAU98542.1      FIWGFGKQGFQCQVCCFVVHKRCHEFVTFSCPG----------ADKGPDTDDPRSKHKFK
KAI4054112.1    FIWGFGKQGFQCQVCCFVVHKRCHEFVTFSCPG----------ADKGPASDDPRSKHKFK
XP_809943.1     ------------------------------------------------------------
XP_845346.1     ------------------------------------------------------------
PWV04907.1      FTPKCLKCGVTFPSPRRTIPPKRWECPTKGCGG----------KLWQPDDASQVCDVCGA
ESS64742.1      VKWHVFRRYQQFRALDQKLKQFCSMGSQYHCEYGVLPVLTGSHWAEVTNQSIDLVEKRRR
AAK64304.1      MTWQVFRRYQQFKALDQSLKQLCSRGSSRHCDYGVIPVLCGSHWTEVTNQSIDLVEKRRR

BAU98542.1      IHTYGSPTFCDHCG------SLLYGLIHQGMKCDTCDMNVHKQCVINVPSLCGMDHTEKR
KAI4054112.1    IHTYSSPTFCDHCG------SLLYGLIHQGMKCDTCMMNVHKRCVMNVPSLCGTDHTERR
XP_809943.1     -----------------------------------------------MSVEYSGYLQKTG
XP_845346.1     -----------------------------------------------MTIDYSGFLDEPS
PWV04907.1      MVARFTRHHCRRCGYLTCSKCLKFKTILTEWSLTEKQRVCRRCAVPAVPVAISGWLLKLG
ESS64742.1      YLEIYLEQLLVPKNLFYVAKTALYKFLHEGEVPVRCGTNVLRPLIGFAAVE---PSRRPE
AAK64304.1      HLEIYLRQLLVPGNVFYVAKTVIYDFLHDGAVPAQHQRRAIRPLIGFATPDSLADLHLEN
                                                .

BAU98542.1      GRIYLKAEVADEKLHVTVRDAKNLIPMDPNGLSDPYVKLKLIPDPKNESKQKTKTIRSTL
KAI4054112.1    GRIYIQAHIDRDVLIVLVRDAKNLVPMDPNGLSDPYVKLKLIPDPKSESKQKTKTIKCSL
XP_809943.1     G---KFYKKNQTRYFELRGPMLCYWKRRPSGPHVS-------------------------
XP_845346.1     G---ASSEVTASRYFEIRGSILYCWTYKPENPGDK-------------------------
PWV04907.1      QRATFFGEKLHMRYFELRGTVLLYGRAKSNGEADEGLTSVSLPKDARRGRGTGSGISTSD
ESS64742.1      ESEDDATTVEESPNPLDISAAGGNLPVTTTFCTTPERGVSN----SVESFSTNRSSGMEE
AAK64304.1      RNGEGSASRLGAASTLNDKSISMLGPSAVGESDRVDDGVGDEEEEPCKSPPGETVESGEE

BAU98542.1      NPQWN----------------------------ESFTFKLKPSDKDRRLSVEIWDWDRTT
KAI4054112.1    NPEWN----------------------------ETFRFQLKESDKDRRLSVEIWDWDLTS
XP_809943.1     -------------------------------------PTGTIDLTNARFVENPKDPRSWT
XP_845346.1     -------------------------------------PLSSIDLTDVHITRDEADRRSWS
PWV04907.1      GANSSSSNSHMNSKNNNNNNNCGSSSSSSNAQPTNSILTGQIDISGAKVMDVAIHPHAFC
ESS64742.1      YDDVPSRED------------------TDNEDIHMPPASPLCLQCNAEFSSMLYPHRCFL
AAK64304.1      CEDLPSADN------------------SEAEDVRVPPPGSLCRQCNAEFSSVLYPHRCFF
                                                             . ..

BAU98542.1      RNDFMGSLSFGVSELMKMPASGWYKLLNQEEGEYYNVPIPEGDEEGNMELRQKFEKAKLG
KAI4054112.1    RNDFMGSLSFGISELQKASVDGWFKLLSQEEGEYFNVPVPPEGSEANEELRQKFERAKIS
XP_809943.1     IEGDHLSKTFTLTADTEEQREAWVREMSKVKPENREQPSTAVQSSGDVHAVTLYTGGR--
XP_845346.1     VQGGKLRKPHMFTAENEEEREVWIEKMAHPNPANTEILRTLEQTSDGDDTAPLCSGGS--
PWV04907.1      IVGPFLPRGYVLSASSQAAKTQWVEAIREQVRKAHQQRGDEEFEDDLESTEKVLYAGSGA
ESS64742.1      CKQRFCRNCLRNVELEE-EVVRVCLQCYENHERHSKKKQTAKDATPETPFSQFKGVTTAP
AAK64304.1      CRQQFCRDCLHPVELEEGEVARSCVQCYENFARKTCKPQYPPTPMASTPILQCAPAGGAL
                                :           .

BAU98542.1      P---AGNKVISPSEDRKQPSNNLDRVKLTDFNFLMVLGKGIFGKGMLSHKKGTKEMYAIK
KAI4054112.1    QGTKVPEEKTTNTVSKFDNNGNRDRMKLTDFNFLMVLGKGSFGKVMLSERKGTDELYAVK
XP_809943.1     -----------------------HKVSLDDFELKATIGAGSFSNVFVAREKSTDKVYAIK
XP_845346.1     -----------------------NRVSLNDFQFAAKIGKGSFSSVYAATEKATGKTYAIK
PWV04907.1      D--------------------KNVNVSMRDFELLTVIGLGSFGRVMRARHKATGKIYALK
ESS64742.1      EILQLHEGN-----------QLRTDVSLSDFELVTTLGRGTFGKVLKVIFRENGQVYALK
AAK64304.1      DPLNLLGSTS----------HVRTDVTLSDFKLVTTVGRGTFGKVMKVIFREDGKVYAMK
                                         :.: **::   :* * *.       :   : **:*

BAU98542.1      ILKKDVVIQDDDVECTMVEKRVLALLDKPPFLTQLHSCFQTVDRLYFVMEYVNGGDLMYH
KAI4054112.1    ILKKDVVIQDDDVECTMVEKRVLALPGKPPFLTQLHSCFQTMDRLYFVMEYVNGGDLMYH
XP_809943.1     EMGKELIQQHNMLSNIAAEKHILQTISHP-FIVSLHYAFETKKCLYLVLDFLPGGELFFH
XP_845346.1     KMEKEVIERYNMIDNISAERLILQKIDHP-FIVSLHYAFQTKGSLYLVMDFLSGGELFFH
PWV04907.1      ILDKDQVVQHHLVPHTNAEKIILSEISHP-FVVKLYYAFQTKRHLILVLEFLCGGELFFH
ESS64742.1      ILNKCIIHKRRMVEYIKEEKDIMASLPFHPYVVTCHFAFQTDYHLFFVLDFLPGGELYSH
AAK64304.1      VLNKCVIHKRRMIEYIREEKNIMSSLPSHPYIVTCHFAFQTDYHLFFVLDYLPGGDMHSR
                 : *  : :   :     *:  ::      ::.  : .*:*   * :*:::: **::  :

BAU98542.1      IQQVGKFKEPQAVFYAAEISIGLFFLHKRGIIYRDLKLDNVMLDSEGHIKIADFGMCKEH
KAI4054112.1    IQQVGRFKEPHAVFYAAEIAIGLFFLQSKGIIYRDLKLDNVMLDSEGHIKIADFGMCKEN
XP_809943.1     LAKEKVFDEYRAKFYCGEIALAIGYLHSLDIIFRDLKPENIVLDEDGHACLTDFGLAKMN
XP_845346.1     LESVSVFDEWRAKFYCGEIALALGYLHAQDIIYRDLKPENAVLDADGHVCLTDFGLAKMD
PWV04907.1      LQRCKRFDENRARFYTAEIGMAVEYIHSKSVLYRDLKPENLVLDREGHVVLTDFGLAKRD
ESS64742.1      IYPKCTLSPVDVRLIIAEVVLALEHLHRYDIVHRDLKPENIVFAADGHLKLTDFGLARMN
AAK64304.1      VYPKLKLTESDVRLYIAELVLALQHLHRHDIAHRDVKLENIVLGEDGHLKLTDFGLARMN
                :     :    . :  .*: :.: .::  .: .**:* :*  :: :**  ::***:.: .

BAU98542.1      MMDGVTTRTFCGTPDYIAPEIIAYQPYGKSVDWWAYGVLLYEMLAGQPPFDGEDEDELFQ
KAI4054112.1    IWDGVTTKTFCGTPDYIAPEIIAYQPYGKSVDWWAFGVLLYEMLAGQAPFEGEDEDELFQ
XP_809943.1     VSNASN-FTFCGTTEYIAPEFLLGQPHGRAVDWWALGILLYEMIEGIPPFFNENSNEMYE
XP_845346.1     VRDACN-FTFCGTPEYIAPEFLLGKPHGKAVDWWSLGILLYEMLEGIPPFYSENVSAMYD
PWV04907.1      VAEDVRTHTFCGTPEYMAPELVKKSGHTTAVDWWSLGAFLYEMVDGMPPFYSPNVAEMYE
ESS64742.1      FSRHRR-YSFVGSPEYLAPETIRGECQSRALDWWSVGVMMYEMLVGSTPFHAANNNDVCN
AAK64304.1      FSRQRR-RSFVGSPEYLPPETIQGKYQTKAVDWWSAGVMLYEMLSGKTPFYSAYNCEIYN
                .       :* *:.:*:.** :  .    ::***: * ::***: * .**       : :

BAU98542.1      SIMEHNVSYP---------KSLSKEAVSTCKGLMTKHPAKRLGCGPEGERDVREHAFFRR
KAI4054112.1    SIMEHNVAYP---------KSMSKEAVAICKGLMTKHPGKRLGCGPEGERDIKEHAFFRY
XP_809943.1     EILKGELKFGDVGGEESGLPVISENAKALLRRLLDRNPQTRLQDLEE----FKKHPFFED
XP_845346.1     KILSSELQFGDGEGGSNNMPQISEEAQDLLRRLLDRNPDTRLQDVEE----LKGHPFFRD
PWV04907.1      MILHKPLVCP---------ARFSPELKSLLGRLLEKDPLKRMTSGEE----FRAHPFFNG
ESS64742.1      NVLNRTLDLT--------APCFTPEAASLIRQLLQRQPKQRLRDAEQ----IKAHPYFAS
AAK64304.1      NVLKAELDLT--------APCFTPEAASLIEQLLQSHPKARLQDAGA----IKAHPYFAS
                 ::   :              :: :       *:  .*  *:         .: *.:*

BAU98542.1      IDREKLENREIQPPFKPKVCGKGAENFDKFFTRG--------------------------
KAI4054112.1    IDWEKLERKEIQPPYKPKACGRNAENFDRFFTRH--------------------------
XP_809943.1     IDWVKLSRREIQPPFRPSS--NILCNFDEDFTSK--------------------------
XP_845346.1     LDWEKLFRREIEPPFRPDG--NALSNFDQEFTSA--------------------------
PWV04907.1      LDFDKLLRREIRPKFVPDVRRNDLRYFDKRFLHESTKIAQLDQPCDPSDPVSRAFEG---
ESS64742.1      LNWSALENKSLPPPIRLNLEGNDTKYFKREFTAEWAVIARPQEVSRATLDMLKKQFSNFV
AAK64304.1      IDWAALEGKKISAPIQLDLMGNDMKYIKWKFTAEWAVIYKPPGVTRATIDLLINRFSNFA
                ::   *  :.: .    .   .    :.  *

BAU98542.1      ------------------------------------------------------------
KAI4054112.1    ------------------------------------------------------------
XP_809943.1     ------------------------------------------------------------
XP_845346.1     ------------------------------------------------------------
PWV04907.1      ------------------------------------------------------------
ESS64742.1      HVPDVGAGVSLPSPSSGKRASLPQSIKADADEVLTVQRLLGIWRLVRVEMTTDDGKIAYP
AAK64304.1      HVSEG--------TPPTPHLPIGQQQEEVSDGVTNPPDITGVWNVVKVEMQTEDGKITHP

BAU98542.1      ---------------------------QPVLTPPDQLVIANIDQSDFEGFSYVNPQFVHP
KAI4054112.1    ---------------------------PPVLTPPDQEVIRNIDQSEFEGFSFVNSEFLKP
XP_809943.1     ---------------------------EPRAGFQEEDGGEHGNIACFSFDGQMGPA----
XP_845346.1     ---------------------------DPPMVQPDDEVVEDKSICGFTFNGRSRPT----
PWV04907.1      -----------------YEFNVHVPTTQPAMEEKRDDIDSHDDDGGDVKDGGGDATHTA-
ESS64742.1      WGSEVCGLLAYFPNGIFTMQFTLSRRPHTGSHFPEQATVDELVEMCNSYVASFGRYQLKA
AAK64304.1      WGSAVCGVLAYFPEGQFSMQLTSYMRPHLRQQFVDRAVREDLVEMCNSYAGSFGKYQIKP
                                                        .         .

BAU98542.1      ILQSAV------------------------------------------------------
KAI4054112.1    EVKS--------------------------------------------------------
XP_809943.1     ------------------------------------------------------------
XP_845346.1     ------------------------------------------------------------
PWV04907.1      ------------------------------------------------------------
ESS64742.1      ESNAIVHCPNGSLFPNPSWNQQKFFVEVSSEKSVGGVAALKLCTPQYNLQEEQLLARTVL
AAK64304.1      GSNIITHRLHGCLCPNLTGSTQKYFFEVR-ERKENGAKVLKLFTACNALPGEDISAQTVL

BAU98542.1      --------
KAI4054112.1    --------
XP_809943.1     --------
XP_845346.1     --------
PWV04907.1      --------
ESS64742.1      TWERADAC
AAK64304.1      TWERTGSC
```

Figure S1: Alignment of PKC amino acid sequences. Multiple sequence alignment using the ClustalW algorithm (https://www.genome.jp/tools-bin/clustalw) was performed using the following sequences: BAU98542.1 protein kinase C alpha [Homo sapiens]; KAI4054112.1 protein kinase C beta [Homo sapiens]; ESS64742.1 protein kinase [Trypanosoma cruzi Dm28c (TcPKC1)]; PWV04907.1 putative serine-threonine kinase [Trypanosoma cruzi TcPKC2]; XP_809943.1 serine-threonine kinase [Trypanosoma cruzi strain CL Brener TcPKC3]; AAK64304.1 zinc finger protein kinase

[Trypanosoma brucei brucei]; XP_845346.1 putative serine-threonine kinase [Trypanosoma brucei brucei TREU927].
