# Supplementary material for: Trypanosoma cruzi DNA Polymerase β Is Phosphorylated In Vivo and In Vitro by Protein Kinase C (PKC) and Casein Kinase 2 (CK2)
Source: Cells. 2022 Nov 21;11(22):3693. doi: 10.3390/cells11223693 (PMC9688435; doi:10.3390/cells11223693)
Supplement: Supplementary file 1 [file cells-11-03693-s001.zip › PKC.FigureS2.pdf]

clustalw.aln

```
CLUSTAL 2.1 multiple sequence alignment


AAH70052.1      ------------------------------------------------------------
KAF8293196.1    ------------------------------------------------------------
NP_587933.1     MSSSSNTSSHRSYGLRRSQRSMNLNRATLLAPPTPSSLYDANNSTSSTSSQKPNTSFTSL


AAH70052.1      ------------------------------------------------------------
KAF8293196.1    ------------------------------------------------------------
NP_587933.1     FGPRKQTTSSPSFSHAAPLHPLSPPSFTHSQPQIQAQPVPRRPSLFDRPNLVSRSSSRLG


AAH70052.1      ---------------------------------------------------------MSF
KAF8293196.1    ------------------------------------------------------------
NP_587933.1     DSPSLSPVAQVANPIHHTAPSPSDVRAFPIHKNASTGVKRSFFSSSMSNGAMSPPSHSPS


AAH70052.1      LSRQQPPPPRRAGAACTLRQKLIFSPCSDCEEEEEEEEEEGSGHSTGEDSAFQEPDSPLP
KAF8293196.1    ------------------------------------------------------------
NP_587933.1     PFLQSSQHIPPSTPAQKLRKKNNFDSFRISNSHISPFASGSFSPFATSSPNFLSTSTPAP


AAH70052.1      PARSPTEPGPERRRSPGPAPGSPGELEED-----LLLPGACPGADEAGGGAEGDSWEEEG
KAF8293196.1    ---------------------------------------------------MSMARAKFS
NP_587933.1     PNSNNANPSTLFSSIPSSRHTTSNHFPSNSAQSSLFSPTARPLTARKLGFASSQTKSAVS
                                                                    . :    .

AAH70052.1      ---------------------------------------------------FGSSSPVKS
KAF8293196.1    ---------------------------------------------------LGVVSFDFT
NP_587933.1     NNHSRNSSKDASFMMKSFIPSNRSHPQTQQNESSLFSDNSMVNSSSNSFSLFPNATLPNP
                                                                   :   :   .

AAH70052.1      PAAPYFLGSSFSPVRCGGPGDASPRGCGARRAGEGRRSPRPDHPGTPPHKTFRKLRLFDT
KAF8293196.1    PTESVDAGMLMYYLKCG-------------------------------FHTFTFQFLFHM
NP_587933.1     PSSELLTTPFQQIKPPSQVFMSTGLLSKQHRPRKNINFTPLPPSTPSKPSTFVRPHSSST
                *:              .                                 **

AAH70052.1      PHTPKSLLSKARGIDSSSVKLRGSSLFTDTEKSGKREFDVRQTPQVNINP----------
KAF8293196.1    FTAIMYWLKFLFLFLYFCVYVVIS--------VSSFLFEIRDKLRAMGHP----------
NP_587933.1     DSPPSPSTPSNTQTDSYFIQRENTPTNHNSIPTIQLEKSSMDFLRFDPPPSAVKTSHNYG
                  .               :    :          .   .  :  :    *

AAH70052.1      ------------------------FTPDSLLLHSSGQCRRRKRTYWNDSCGEDMEASDYE
KAF8293196.1    ------------------------AMDAADREDSHAGCFSDSVGMQTYSLNTSLG-----
NP_587933.1     LPFLSNQRCPATPTRNPFAFENTVSIHMDGRQPSPIKSRNNNQMSFAMEEEADVSQPSSS
                                                 *   .   .      .   .:

AAH70052.1      LEDETRPAKRITITESNMKSRYTTEFHELEKIGSGEFGSVFKCVKRLDG-CIYAIKRSKK
KAF8293196.1    --------------AMHIMSRLNNEMSFVEDTASGSFGVVKKGVLDLDR-QCYAVKQTKR
NP_587933.1     SFTLSFPSALTSSKVSSSTSHLLTRFRNVTLLGSGEFSEVFQVEDPVEKTLKYAVKKLKV
                                   *:  ..:  :   .**.*. * :    ::    **:*: *

AAH70052.1      PLAGSVDEQNALREVYAHAVLGQHSHVVRYFSAWAEDDHMLIQNEYCNGGSLADAISENY
KAF8293196.1    VISGEGDLQQRLQEVYTLSAC-SHPNVLRYFDGWVEDRAVFVRTEWLSDGSVADFDRP--
NP_587933.1     KFSGPKERNRLLQEVSIQRALKGHDHIVELMDSWEHGGFLYMQVELCENGSLDRFLEEQG
                 ::*  : :. *:**    .   * :::. :..* ..  : :: *  ..**:

AAH70052.1      RIMSYFKEAELKDLLLQVGRGLRYIHSMSLVHMDIKPSNIFISRTSIPNAASEEGDEDDW
KAF8293196.1    -----FSEALLRSVIHQIASALHWLLCHHITHRDVKPENILARKMEDGTYTFKLADFG--
NP_587933.1     Q-LSRLDEFRVWKILVEVALGLQFIHHKNYVHLDLKPANVMITFEGT----LKIGDFG--
                     :.*  : .:: ::. .*:::     .* *:** *::           : .* .

AAH70052.1      ASNKVMFKIGDLGHVTRISSPQVEEGDSRFLANEVLQENYTHLPKADIFALALTVVCAAG
KAF8293196.1    LARPLFRDRPNTGEEFRGTND--DDGDRRYLSPEAFAISEFSQQKGEADVYALGASCVEL
NP_587933.1     --------MASVWPVPRGMER---EGDCEYIAPEVLANHLYDKP-ADIFSLGITVFEAAA
                          .     *  .    :** .::: *.:         .:    .: .  .

AAH70052.1      AEPLPRNGDQWHEIRQGRLPRIPQVLSQEFTELLKVMIHPDPERRPSAMALVKHSVLLSA
KAF8293196.1    MGGDPS------LVRNGCYTGNFHIYSAELQNLVQWMTRLDPQERPDAFMVALLTVDP-A
NP_587933.1     NIVLPDNGQSWQKLRSGDLSDAPRLSSTDNGSSLTSSSRETPANSIIGQGGLDRVVEWML
                    *        :*.*  .   :: * :  . :    :  * .   .       *

AAH70052.1      SRKSAEQLRIELNAEKFKNSLLQKELKKAQMAKAAAEERALFTDRMATRSTTQSNRTSRL
KAF8293196.1    LKSTRGFARRMAAIEDLRASVAELEKKVAETNATEKEEGDA-------------------
NP_587933.1     SPEPRNRPTIDQILATDEVCWVEMRRKAGAIIYEGIHGSSSNPQGDQMMEDWQVNV----
                  ..             . .  : . * .       .

AAH70052.1      IGKKMNRSVSLTIY
KAF8293196.1    --------------
NP_587933.1     --------------
```

Figure S2: Alignment of Wee1 amino acid sequences. Multiple sequence alignment using the ClustalW algorithm (https://www.genome.jp/tools-bin/clustalw) was performed using the following sequences: AAH70052.1 Wee1 homolog (*S. pombe*) [*Homo sapiens*]; NP_587933.1 protein kinase Wee1 [*Schizosaccharomyces pombe*]; KAF8293196.1 putative Wee1-like protein kinase [*Trypanosoma cruzi*].
